# Polymyxin sensitivity/resistance cosmopolitan status, epidemiology and prevalence among O1/O139 and non-O1/non-O139 *Vibrio cholerae*: A meta-analysis

**DOI:** 10.1016/j.imj.2023.11.004

**Published:** 2023-11-21

**Authors:** Bright E. Igere, Hope Onohuean, Declan C. Iwu, Etinosa O. Igbinosa

**Affiliations:** a Department of Biological Sciences, Microbiology Unit, Dennis Osadebay University, Asaba 320242, Nigeria; b Biotechnology and Emerging Environmental Infections Pathogens Research Group (BEEIPREG), Department of Biological Sciences, Microbiology Unit, Dennis Osadebay University, Asaba 320242, Nigeria; c Biopharmaceutics unit, Department of Pharmacology and Toxicology, School of Pharmacy, Kampala International University Ishaka-Bushenyi Campus, Ishaka-Bushenyi 10101, Uganda; d Department of Microbiology, University of Pretoria, Pretoria 0002, South Africa; e Department of Microbiology, Faculty of Life Sciences, University of Benin, Benin 300213, Nigeria

**Keywords:** Biotyping scheme, Environmental nonO1/nonO139 *V. cholerae*, Clinical O1/O139 *V. cholerae*, PB-sensitive/PB-resistant strains, Global epidemiological relevance, Polymyxin B

## Abstract

Resistance/sensitivity to polymyxin-B (PB) antibiotic has been employed as one among other epidemiologically relevant biotyping-scheme for *Vibrio cholerae* into Classical/El Tor biotypes. However, recent studies have revealed some pitfalls bordering on PB-sensitivity/resistance (PBR/S) necessitating study. Current study assesses the PBR/S cosmopolitan prevalence, epidemiology/distribution among O1/O139 and nonO1/nonO139 *V. cholerae* strains. Relevant databases (Web of Science, Scopus and PubMed) were searched to retrieve data from environmental and clinical samples employing the Preferred Reporting Items for Systematic Reviews and Meta-Analyses (PRISMA). Random-effect-model (REM) and common-effect-model (CEM) of meta-analysis was performed to determine prevalence of PBR/S *V. cholerae* strains, describe the cosmopolitan epidemiological potentials and biotype relevance. Heterogeneity was determined by meta-regression and subgroup analyses. The pooled analyzed isolates from articles (7290), with sensitive and resistance are 2219 (30.44%) and 5028 (69.56%). Among these PB-sensitive strains, more than 1944 (26.67%) were O1 strains, 132 (1.81%) were nonO1 strains while mis-reported Classical biotype were 2080 (28.53) respectively indicating potential spread of variant/dual biotype. A significant PB-resistance was observed in the models (CEM = 0.66, 95% CI [0.65; 0.68], *p*-value = 0.001; REM = 0.83 [0.74; 0.90], *p* = 0.001) as both models had a high level of heterogeneity (*I*^2^ = 98.0%; ${}_{df=33}^{2}=1755.09$,Qp=2.4932). Egger test (*z* = 5.4017, *p* < 0.0001) reveal publication bias by funnel plot asymmetry. The subgroup analysis for continents (Asia, Africa) and sources (acute diarrhea) revealed (98% CI (0.73; 0.93); 55% CI (0.20; 0.86)), and 92% CI (0.67; 0.98). The Epidemiological prevalence for El tor/variant/dual biotype showed 88% CI (0.78; 0.94) with O1 strains at 88% CI (0.78; 0.94). Such global prevalence, distribution/spread of phenotypes/genotypes necessitates updating the decades-long biotype classification scheme. An antibiotic stewardship in the post antibiotic era is suggestive/recommended. Also, there is need for holistic monitoring/evaluation of clinical/epidemiological relevance of the disseminating strains in endemic localities.

## Graphical abstract

**Figure:**
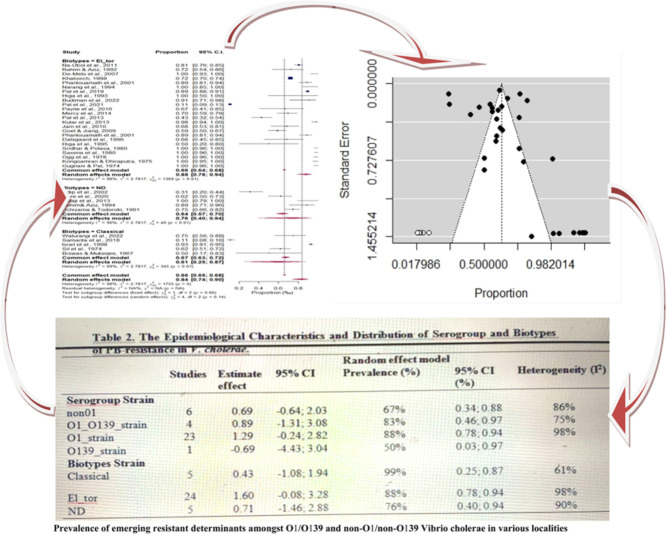

## 1 Introduction

The notable disappearance and re-establishment of *Vibrio cholerae* with remarkable reports of its changing nature since 1992 has impacted their biotype classification scheme. Over the years, the cholera *Vibrio* biotyping has been based on both time and place of first isolation, hence the biotype names are Classical, El Tor, Bengal, and Calcutta [1]. However, sensitivity and resistance to polymyxin B (PB) among other methods have been employed as an epidemiologically relevant scheme for biotyping *Vibrio* members into Classical and El Tor strains, since these are the 2 major biotypes globally implicated and distributed in outbreak reports. PB sensitive strains are biotyped as Classical while PB resistant strains are biotyped as El Tor *V. cholerae* based on a specified concentration of PB [1,2]. Over the years, diverse investigators have applied this biotyping strategy, which has become one major epidemiologically relevant biomarker for *V. cholerae* biotyping scheme [3], [4], [5]. However irrespective of the concentration of PB applied for susceptibility testing, there had been reports of some sensitive El tor *V. cholerae* strains creating a potential indeterminate situation for the biotyping scheme. Some further studies have revealed the emergence of PB sensitive El tor *V. cholerae* strains in addition to diverse biotyping dynamics and dual and/or atypical phenotype [3,^4^,[6], [7], [8], [9], [10], [11], [12], [13], [14], [15]. Suffice it to say that the Lipopeptides antibiotics (polymyxin B, daptomycin, surotomycin, and colistin) have been routinely used in the management of enteric potential pathogens following the Clinical Laboratory Standard Institute (CLSI) guidelines [16]. Resistance to PB by *V. cholerae* strains have also been affirmed in reports, which thrive in the environment of agent by remodeling it's surfaces lipopolysaccharide (LPS) [6,[17], [18], [19], [20]. One surface enzyme expressed by *V. cholerae* with such resistance character is the Alm-EFG which plays a pivotal role in peptide-bound antibiotic resistance. In addition, some investigators have also associated PB resistance to peptide-bound with phosphoethanolamine modification of lipid A [21] predominantly observed among the El Tor *V. cholerae* strains.

Although PB has been used in management of enteric potential pathogen, it is not a commonly employed antibiotic for the treatment of vibriosis casesdue to its potential to cause slight nephrotoxic and neurotoxic effects on human; however, it may be applied as a last source therapy. Polymyxins are highly effective against Gram-negative organisms due to their structural architecture and cationic diaminobutyric domains which interact intrinsically with the negatively charged bacterial lipopolysaccharides (LPS) domain, which results a membrane-hydrophobic surface complex. Further surface accumulation of the polymyxin, culminates a quick pores formation, which results cell lysis [22], [23], [24]. Its resistance is described to be associated with reduced porin expression or modification of LPS architecture [22], [23], [24], [25], [26] where *pmrA* and *pmrB* are associated resistant genes. Resistance to PB has also been reported to be implicated with plasmid (plasmid-encoded *mcr*-1 gene) which encodes a transferase gene or enzyme (phosphoethanolamine transferase) at the lipid A moity.

According to the study of Han and Khie in 1963 and the guideline of CLSI [27], a modified disc diffusion method involves inoculating a standardized test strain saline culture onto M-HA for 4–5 hours at 35 °C, while a 50–200 units of PB disk was placed on confluent growth on medium surface and incubated overnight at 37 °C. A sensitive report shows an inhibition zone range of 12–15 mm whereas a resistance report shows an inhibition zone range of 1–4 mm. of inhibition [17,28]. The observation of El Tor sensitive and/or Classical resistant strains to PB and a reliable estimation of the prevalence/epidemiology possess potential clues or need unto updating the biotyping scheme, accessing resistance mechanism and arouse the need to determine resistance status of *V. cholerae* strains. Such approach would also impact development of effective prevention strides as well as implementing public health programs. It is to this end, this present study determines the PBR/S cosmopolitan prevalence, epidemiology/distribution among clinical and environmental (O1/O139 and nonO1/nonO139 *V. cholerae*) strains: a meta-analysis while employing the Preferred Reporting Items for Systematic Reviews and Meta-Analyses (PRISMA).

## 2 Methods and search strategies

Applying the Preferred Reporting Items for Systematic Reviews and Meta-Analyses (PRISMA) [6,29], our study reports the meta-analysis of studies that have revealed PB resistance and susceptibility among *V. cholerae*. We retrieved scientific evidence on PB resistance and susceptibility for *V. cholerae* strains from PubMed, Web of Science (WOS), SCOPUS electronic databases covering January 1980 and November 2021 on June 27, 2022 at 10:18 GMT+2. The study search terms include “prevalence OR occurrence AND Polymyxin resistance AND Polymyxin sensitive AND Environmental Sample AND non01/non0139 *V. cholerae* OR Clinical AND O1/O139 *V. cholerae*”. The datasets were merged on RStudio versions 4.0.5 using bibliometrix R package [30]. Duplicates were removed and variables were normalized using the ScientoPy and fBasics R-packages [6,31]. Thereafter, review of all retrieved articles and reference lists was done manually to add any pertinent articles independently by 2 reviewers (I.B.E. and H.O.) and double check by exchanging the outcomes among the reviewers while discrepancies were resolves by consensus.

### 2.1 Study eligibility and inclusion/exclusion criteria

All peer-reviewed related articles of both primary and secondary research reports in the various databases that meet the objective of the study were eligible for inclusion. Specifically:

1. Studies that use the traditional phenotypic technique to isolate and identified *V. cholerae* from both environmental and clinical samples using: (i) standard microbiological techniques, (ii) polymerase chain reaction (PCR) genotypic techniques, and (iii) other molecular biology techniques such as whole/partial genome sequencing and MALDI-TOF mass spectrometry for detecting antibiotic sensitive/resistance.
2. Studies that reported the prevalence/occurrence of sensitivity and resistance to PB by *V. cholerae*, the 2 major biotypes and any avirulent/atypical or variant strain.
3. Reports that explicitly states the overall number (population) of samples examined and the number of samples that tested positive for the presence of resistance genes among *V. cholerae*.
4. Written in English-language, full publications of peer-reviewed article with sufficient analyzable data.

**Fig. 1:**
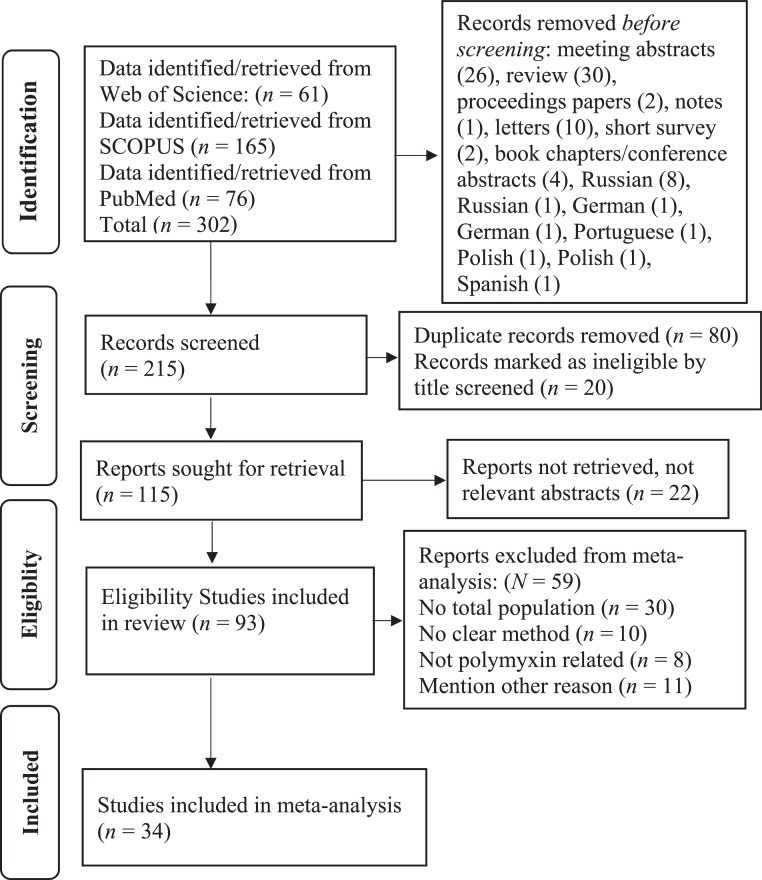
Schematic representation and flow diagram for selecting studies.

We included countries, continent, study concepts and excluded articles not in English and other reasons. The search was further refined by language to include only English language documents; hence 8 documents from Russian and 1 from German were removed to become 52 documents result from Web of Science Core Collection. In Scopus database, 165 documents were recovered with TITLE-ABS-KEY using TITLE-ABS-KEY (vibrio AND cholerae AND polymyxin AND sensitive AND resistance) AND (LIMIT-TO (DOCTYPE, "ar")) AND (LIMIT-TO (LANGUAGE, "English")). Excluded document type includes review/preview (30), Letter (10), Short Survey (2), Book Chapter/notes (1) and Conference Paper (4), meeting abstracts (26), proceedings papers (2) which reduced articles to 90 documents. A further exclusion employed languages such as Russian (1), German (1), Polish (1) which reduced the documents to 87 documents. The PubMed articles extracted 76 documents. The entire retrieved documents were combined as (PubMed = 76, Scopus = 87, WOS = 52) to make a total = 215 using > ABC< −merge Db Sources (WOS, Scopus, PubMed, remove duplicated = T). These documents were checked by IBE, OH and ICD to remove 80 duplicated and twenty records marked as ineligible by title screened (*n* = 20) documents using > write.xlsx (ABC, file = "com.xlsx") to make a total of com. = 115. Other reports not retrieved, due to irrelevant abstracts were 22 (*n* = 22), bringing the total eligible documents to 93. However, 59 other reports were excluded from the meta-analysis (*N* = 59), which made studies included for meta-analysis to be 34 as shown in Figure 1 below.

### 2.2 Data extraction and relevant indices

The names of the first authors, publication year, *V. cholerae* strain, biotypes, PB antibiotic sensitive/resistance, the total number of samples, the number of positive samples, the country of study, the sample source studied, the experimental methods, and the PB-sensitive/resistance breakpoints were identified and extracted from the qualified articles results, figures, tables, and discussions after applying the inclusion and exclusion criteria.

### 2.3 Conceptual background of study

The various articles/documents search and analyzed from the recovered details were guided by previous works of Safa et al. [5,11], and Ahmadi [32] which were further extended in the study of Igere and his colleagues [3,^4^,17]. These authors discussed the prevalence of PB resistant strains, epidemiological relevance of the *V. cholerae* biotyping scheme, reports on the resistance and susceptibility of the strains to the lipopeptidal and/or cataionic antibiotics and the occurrence of variant strains. Such studies also describe the trend in reports as it continues to reveal PB-susceptibility and PB-resistance and its relative associated genes, which may either be chromosomal, LPS modification and/or extrachromosomal (plasmid). The current study investigators were also able to expressly note that the observations in the studied articles would be applicable while analyzing articles indices; measure potential activities; inform surveillance/intervention program design and serve as future guide to monitor potential occurrences. These were carefully considered to ensure appropriateness and focus on the study aim/objective.

### 2.4 Study analysis and software

The *V. cholerae* PB-sensitive/resistance prevalence from the qualified articles were calculated using the raw proportions, while the Wilson method was used to create 95% confidence intervals (CIs). The estimate of summary effect size (weighted average proportion), was used to calculate the pooled effect size based on the individual effect sizes and their sample variances through the argument method = "DL" (using the restricted maximum-likelihood estimator). Since the mean proportion across studies was < 0.025, the logit transformation was used to obtain the pooled prevalence in order to enhance the statistical characteristics [30]. Using sensitivity, influence meta-regression analysis of sampled sources was used to assess heterogeneity and homogeneity effects across the studied samples. The forest plot for the overall analysis and mixed effects model for between-study variance of subgroups (continents, East Africa and epidemiological characteristics of biotype) was analyzed. In order to measure publication bias, funnel plots were created in accordance with Egger's test for asymmetry. The rank correlation test by Kendall's model was then used to determine the significance of the bias. The statistical program R 4.0.5 packages was used to conduct all analyses with 2-tailed of *p*-values < 0.05 level of significance [30]. Further, heterogeneity between studies was quantified using *Q*-statistic and *I*^2^-statistics at a significant heterogeneity threshold [33], [34], [35].

## 3 Results of study

### 3.1 Included studies characteristics

The characteristics for meta-analysis of included studies focused on global polymyxin sensitive/resistance among *V. cholerae* strains, while documents were checked by IBE, OH and ICD for quality and specificity as presented in Table 1. Details shown include authors, year of assessment, Sources of isolates (outbreak/disease cases/food types/environment/water etc.) total number of recovered strains, cases/reports of sensitive strains, cases/reports of resistant strains, reporting continents, reporting countries, strain types in the form of O1, O139, nonO1/nonO139, etc. and the specific biotypes observed in the various studies. It is important to note that analyzed studies employed the use of polymerase chain reaction for gene detection while Kirby–Bauer disk diffusion & broth dilution test were applied in antibiotic susceptibility testing; hence they were not included in the analysis details.

**Table 1:** Characteristics of studies on global polymyxin resistance/sensitive *V.* cholerae meta-analysis.

Authors year	Total	CasesS	CasesR	Countries	Continents	Source	Strains	Biotypes
Na-Ubol et al. [36]	330	64	266	Thailand	Asia	Acute_environment	O1_strain	El_tor
Ndip et al. [37]	55	38	17	Cameroon	Africa	Environmental	nonO1	ND
Rahim & Aziz [38]	29	8	21	India	Asia	Environmental	nonO1	El_tor
De-Melo et al. [39]	104	0	104	Brazil	South America	Acute_environment	O1_strain	El_tor
Khatovich [40]	1479	413	1066	Ukraine	Europe	Acute_environment	O1_strain	El_tor
Phantouamath et al. [41]	99	11	88	Lao PDR	Asia	Outbreak	O1_strain	El_tor
Narang et al. [42]	44	0	44	Sevagram	Asia	Outbreak	O1_strain	El_tor
Pal et al. [43]	1200	127	1073	India	Asia	Outbreak	O1_O139_strain	El_tor
Iwanaga et al. [44]	99	11	88	Japan	Asia	Outbreak	O1_strain	El_tor
Budiman et al. [45]	51	9	42	Indonesia	Asia	FruitsVegetables	O1_O139_strain	Classical/EL_tor
Igere et al. [17]	61	23	38	South Africa	Africa	Environmental	nonO1	ND
Nayak et al. [46]	1200	1070	130	India	Asia	Outbreak	O1_strain	El_tor
Samanta et al. [47]	260	231	29	India	Asia	Outbreak	O1_strain	Classical
Payne et al. [48]	15	5	10	India	Asia	Outbreak	O1_O139_strain	El_tor
Mercy et al. [49]	76	23	53	Kenya	Africa	Outbreak	O1_strain	El_tor
Pal et al. [50]	82	47	35	Kenya	Africa	Outbreak	O1_strain	El_tor
Balaji et al. [51]	31	0	31	India	Asia	Acute	O1_strain	ND
Kutar et al. [52]	119	2	117	India	Asia	Outbreak	O1_strain	El_tor
Jain et al. [53]	41	13	28	India	Asia	Acute	O1_strain	El_tor
Goel & Jiang [54]	114	47	67	India	Asia	Outbreak	O1_strain	El_tor
Israil et al. [55]	624	1	580	Romania	Europe	Outbreak	O1_strain	Classical
Dalsgaard et al. [56]	19	6	13	Guinea Bissau	Africa	Outbreak	O1_strain	El_tor
Higa et al. [57]	8	4	4	India	Asia	Outbreak	O139_strain	El_tor
Jagadeeshan et al. [58]	62	26	36	India	Asia	Environmental	nonO1_ O139_strain	ND
Uchiyama & Todoroki [59]	118	30	88	Japan	Asia	Environmental	nonO1	ND
Sridhar & Polasa [60]	189	0	189	India	Asia	Outbreak	O1_strain	El_tor
Saxena et al. [61]	209	0	209	India	Asia	Outbreak	O1_strain	El_tor
Ogg et al. [62]	179	0	179	USA	North America	Outbreak	O1_strain	El_tor
Kongsamran & Dhiraputra [63]	147	0	147	Thailand	Asia	Acute	O1_strain	El_tor
Sil et al. [64]	79	30	49	India	Asia	Acute	nonO1	Classical
Gugnani & Pal [65]	188	0	188	India	Asia	Acute	O1_strain	El_tor
Biswas & Mukerjee [66]	6	3	3	India	Asia	Acute	O1_strain	Classical

S = Susceptible, R = Resistance, ND = Not determined.

Based on the exclusion/inclusion criteria, a total of 302 articles were recovered from the 3 databases. Upon removal of duplicates and nonrelevant documents, it yielded 93 eligible articles which were reviewed and 34 full-texts studies were meta-analyzed for PBS/R *V. cholerae* (Fig. 1).

### 3.2 Study quality

Quality scores of study was interpreted using the following keyword: total score ≥18 = low bias; 14–18 = moderate bias; and ≤13 = high bias for all included documents/articles which showed an overall quality percentage of 11.77% (4) moderate bias and 88.24% (30) low bias among 34 included articles/documents revealing the appropriateness in quality of assessed/included documents Supplemental Table S1a, b.

### 3.3 Study quality assessment for prevalence studies appraisal

The authors assessed various studies using the assessment tool for prevalence/epidemiological studies to appraise the qualitative/quantitative feature and also assess the risk of bias related to the purpose of the study, method of data collection/retrieval, methods applied during analysis and appropriateness and/or soundness of statistical instruments employed on studies. IBE performed the quality appraisal of all qualitative/quantitative studies and quality appraisal of prevalence/epidemiological check on retrieved studies using 11 major comments and 3 response points (0,1,2) (Supplemental Table S1a, b). Since the study covers epidemiology/prevalence, status of PB sensitive/resistance reports, all studies that report the use of standard disc diffusion test and Polymerase chain reaction were employed regardless of their scores. In the meta-analysis of recovered documents, authors noted the qualitative studies hence there is likelihood of null/low bias scores.

### 3.4 Study outcome

Among the 34 studied articles, 23 of them reported studies on O1 serogroup, 4 of them reported O1/O139, one of them reported O139 serogroup while 6 of them reported cases of nonO1 serogroup members. From the 34 eligible articles/reports, 18 were from outbreaks source, 6 were from acute diarrhea cases, 5 were from environmental sources, 3 were from acute diarrhea and environment while 2 were from fruits and vegetables sources. The biotypes determined during the assessment were El tor (24), Classical (5) while 5 of the investigators fail to determine the biotype of the strains however these strains harbors some El tor genetic markers showing that they are El tor strains [3]. In addition, most of the studies did not reveal the serotype; however, Ogawa strains were mainly reported from the pooled serotype (Table 1) while some investigators reported Inaba.

### 3.5 Global prevalence estimate of polymyxin resistance/sensitive on *V. cholerae*

The pooled/total analyzed isolates were 7290, while sensitive and resistance strains were 2219 (30.44%) and 5071 (69.56%). Among the PB-resistant strains reported, the Classical biotype strains are 682 (13.45%) while the El tor strains are 4148 (81.80%). Strains such as O1 strains, nonO1 strains and O1/O139 shows 3675 (72.5%), 237 (4.67%) and 1112 (21.9%) respectively, while others are O139 strains. Among the PB-sensitive strains, more than 1944 of them were O1 strains; 4 of them were O139 only; 132 of them were nonO1 strains (Table 1). It is worthy of note that most of these PB-sensitive strains were El tor biotype (a total of 1853 strains) which shows that 83.51% of the investigators isolated strains were PB-sensitive. This is an indication that there is a high spread/distribution of PB-sensitive *V. cholerae* strains which are El tor biotype and/or variant/dual biotype strains.

**Fig. 2:**
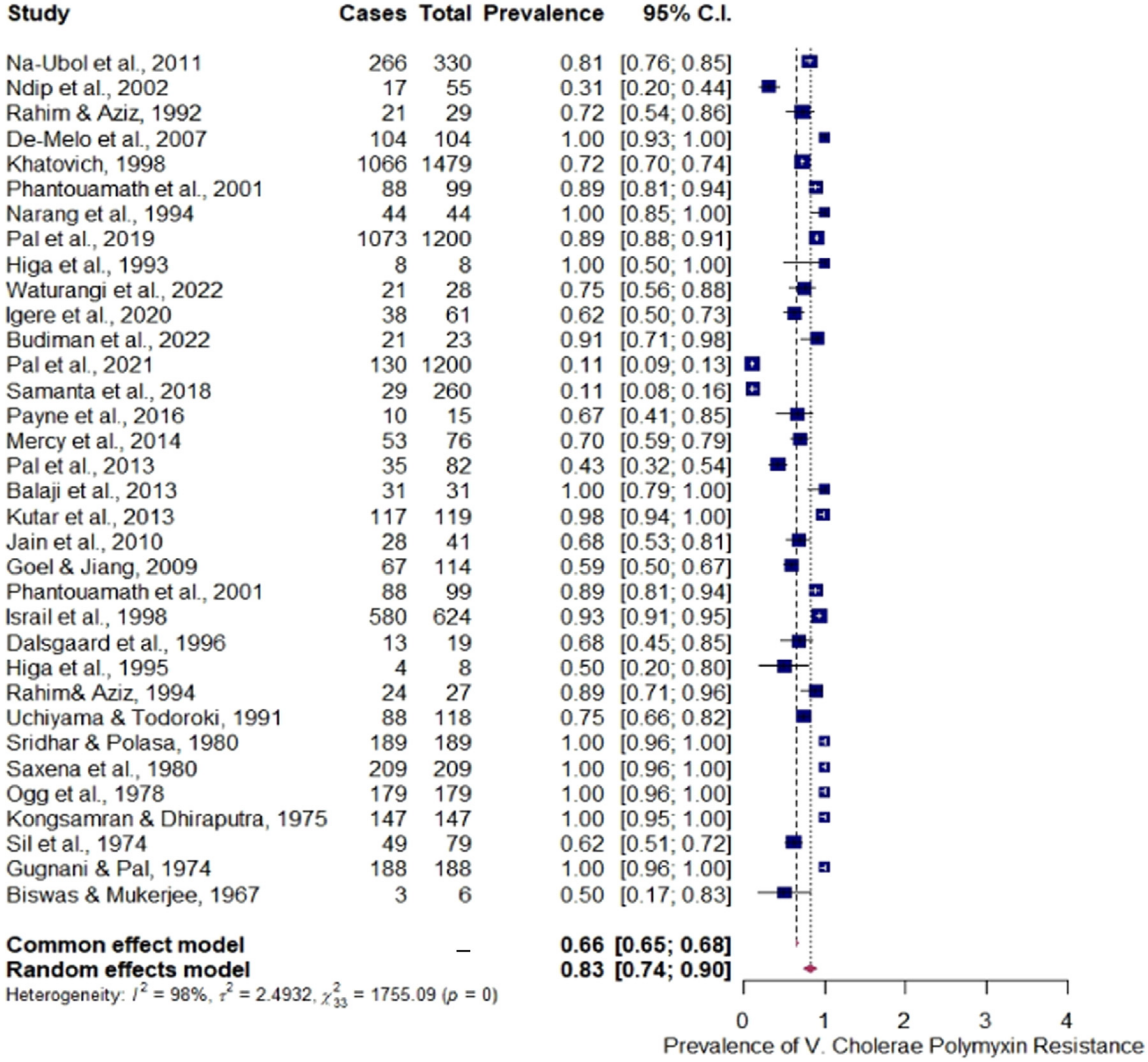
Forest plot for the prevalence polymyxin resistance in *V.*cholera [17,36–66].

The Figure 2 below shows the forest plots on the prevalence of PB resistance among *V. cholerae* as pooled from reports of various authors. A significant PB-resistance was observed in the (common-effect-models and random-effect-models) models (CEM = 0.66, 95% CI [0.65; 0.68], *p*-value = 0.001; REM = 0.83 [0.74; 0.90], *p* = 0.001). Both models also show a high level of heterogeneity (*I*^2^ = 98.0%; ${}_{df=33}^{2}=1755.09$,Qp=2.4932), which indicates that the overall prevalence of meta-analysis revealed a substantial heterogeneity.

**Fig. 3:**
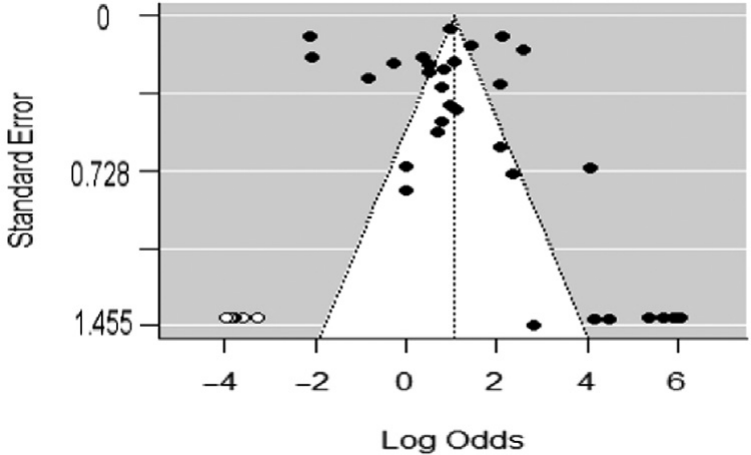
Publication bias test for the polymyxin resistance prevalence in *V.* cholerae.

Also, the Funnel plot (Fig. 3) asymmetry depicts a significant level of publication bias as revealed by the Eggers’ test indicating the presence of publication bias/funnel plot asymmetry *z* = 5.4017, *p* < 0.0001 Limit Estimate (as sei −> 0): *b* = −0.2778, 95% CI: (−1.1812, 0.6257), whereas there are 292 (4.01%) strains of undetermined (ND) biotypes. It is important to note that the PB-sensitive/resistance *V. cholerae* is distributed across the continents of Asia, Africa, North/South America and Europe (ie, sensitive/resistance 25; 73.53%), (5; 14.71%) respectively.

In addition, the biotypes observed during the study were El tor, Classical and undetermined members as shown from the analysis. Among the 34 articles included in the study, 24 (70.59%) investigators reported studies on El tor strains while 5 (14.71%) articles reported Classical biotype. Some of the investigators [5 (14.71%)] report did not describe the identified biotypes which are tagged as undetermined (UD).

### 3.6 Subgroup analysis

The subgroup analysis on prevalence of polymyxin resistance among *V. cholerae* strains among various continent (Asia, Africa, South America, Europe, and North America) categorical variables shows prevalence estimate of 85.45% CI (0.73; 0.93), 54.91% CI (0.19; 0.86), 99.52% CI (0.69; 0.99), 85.33% CI (0.32; 0.98), 99.72% CI (0.80; 1.00) respectively in Supplemental Figure S1. The subgroup analysis by isolates sources, reveal variation in acute environment, environmental, outbreaks, fruits vegetables, acute diarrhea as 90% CI (0.48; 0.99), 68% CI (0.28; 0.92), 84% CI (0.68; 0.93), 85% CI (0.26; 0.99),92% CI (0.67; 0.98) respectively as detailed in Supplemental Figure S2. The occurrence of the sub-categories given by strain and biotype epidemiological characteristics is depicted in Table 2. The El tor strains effect estimate was 1.60, with an *I*^2^ of 98% as reported by 24 articles. Although the classical strains have the highest prevalence of 99%, with an *I*^2^ of 61% presented in 5 studies, the prevalence of the El tor biotypes remains evident. On the other hand, the serogroup that shows high prevalent to PB-resistance includes O1 to be more frequent with 98% prevalence, estimated effect of 1.29 in 23 studies and nonO1, has a prevalence of 86% with estimated effect of 0.69 in among 6 studies (details are presented in Table 2, Supplemental Figs. S3 and S4).

**Table 2:** The epidemiological characteristics and distribution of serogroup and biotypes of PB-resistance in *V. cholerae*.

	Random effect model					
	Studies	Estimate effect	95% CI	Prevalence (%)	95% CI	Heterogeneity (*I*^2^)
**Serogroup strain**						
non01	6	0.69	−0.64; 2.03	67%	0.34; 0.88	86%
O1_O139_strain	4	0.89	−1.31; 3.08	83%	0.46; 0.97	75%
O1_strain	23	1.29	−0.24; 2.82	88%	0.78; 0.94	98%
O139_strain	1	-0.69	−4.43; 3.04	50%	0.03; 0.97	−
**Biotypes strain**						
Classical	5	0.43	−1.08; 1.94	99%	0.25; 0.87	61%
El_tor	24	1.60	−0.08; 3.28	88%	0.78; 0.94	98%
ND	5	0.71	−1.46; 2.88	76%	0.40; 0.94	90%

It is worthy of note that all mined studies for the meta-analysis were reporting from diverse regions, countries and continents which covers Asia, Africa, North/South America and Europe. From the various recovered documents, it was observed that majority of the reports were from Asia (25; 73.53%) which is followed by Africa (5; 14.71%). Other reporting continents such as North/South America and Europe had 2 investigators documents and/or reports each.

### 3.7 Source of heterogeneity analysis for PB-resistance in *V. cholerae* prevalence estimate

The potential sources of heterogeneity seen in the visual forest plot were investigated using univariant meta-regression analyses. The result indicates that subgroups analysis of continents, source, strains, and biotypes where not significantly associated with the heterogeneity (*p* = 0.0553; *R*^2^ = 0.00%), (*p* = 0.7221; *R*^2^ = 0.00%), (*p* = 0.3063; *R*^2^ = 0.00%), and (*p* = 0.1383; *R*^2^ = 0.00%). However, the sampling method applied in some studies may account for a significant total variability *p* < 0.0001, *R*^2^= 53.18%.

## 4 Discussion

The cholera pandemic has continuously remained recalcitrant since over 300 years (1817–2022) of reports in various continents (such as Africa, Europe, and Asia) as it has spread within diverse regions, districts and municipalities. The biotype classification scheme of the culprit potential pathogen (*V. cholerae*), has remained susceptible to PB or resistant to PB which characterizes the strain to either classical and/or El Tor. Although the epidemiological relevance of applying this strategy has been retained for several decades, its sustainability, dependability and authenticity has been abridged by recent reports of emerging dual biotype strains [4,^5^,17] and variant strains. In the earlier study of Mathur and Waldor [67], it was reported that the outer membrane protein unit (ompU) of *V. cholerae* does confer resistance to PB-Sulfate, while mutant ompU or absence of ompU enhances sensitivity to PB. Such observation implies that strains with positive-porin or ompU imparts resistance to PB or cationic antibacterial agent or the lipopeptidal antibiotics via a familiar mechanism.

It is also evident that susceptibility to polymyxin possesses potential for relevant dynamics among various *V. cholerae* and other enterocyte infecting potential pathogens as previously reported by Mathieu-Denoncourt and Duperthuy [68]. Their *V. cholerae* secretome study which analyses the role of PB sub-inhibitory concentration revealed that there is an undetermined large quantity of extracellular proteins expression owning to either the presence or absence of PB. In another study, it was reported that the *V. cholerae* strains does release exogenous proteins when exposed to some PB or lipopeptidal antibacterial agents which expedite adaptation and/or resistance to host environment [69]. In an earlier study, some investigators posit that the outer membrane vesicle (OMV) released from El Tor *V. cholerae* (O1 strain) in the presence of PB consist large size of biofilm-associated extracellular matrix protein (BAP1) (a modified OMV) which aids infectivity and translocation of effector proteins [70]. Other related studies in Odisha, India, also reported the observation of some O1 and Ogawa serotype *V. cholerae* El tor variant strains in outbreaks which are sensitive to PB. Such variant strains with PB-sensitivity indices are shown to be replacing the wild type or proto-typical resistant El Tor *V. cholerae* strains [71]. Some studies reported a co-resistance mechanism of doxycycline and a member of the lipopeptide antibiotic (colistin) [72]. From the foregoing, such reports suggest that the status of polymyxin resistance and sensitivity as a biotyping scheme is questionable. Due to the aforementioned, there is a current growing need for development of novel therapeutic formulations and adroit policy implementation with surveillance monitoring strides for global effective usage of recommended antibiotics in order to prevent further rise and spread of multiple antimicrobial resistance determinants.

Reviewing these aforementioned reports systematically and meta-analysis, a high PB sensitive prevalence was observed among *V. cholerae* especially among the El tor biotype strains (Table 1) with majority of reports documented in Asian countries (73.53%) and African countries (14.71%). Such observation reveals the emerging tendency of *V. cholerae* strains, distribution and epidemiological relevance of such strain and also highlights the need for review of the biotyping scheme, regional antibiotic susceptibility profiling prior to antibiotic administration. A high level of heterogeneity (*I*^2^ = 98.0%; ${}_{df=33}^{2}=1755.09$,Qp=2.4932) was also observed which indicates high prevalence of such PB sensitivity El tor strains especially among the O1 serogroup members (Fig. 2). Also, the Funnel plot (Fig. 3) of the PB resistance prevalence among *V. cholerae* depicts the test of publication bias. Such publication bias was further substantiated by the Eggers’ test which reveals the presence of publication bias/funnel plot asymmetry *z* = 5.4017, *p* < 0.0001 Limit Estimate (as sei −> 0): *b* = −0.2778, 95% CI (−1.1812; 0.6257).

The various genes that encourage sensitivity to PB among *V. cholerae* were also analyzed by various investigators [67], [68], [69], which are genetic components present in the chromosome of *V. cholerae* strains. In addition, the report of PB resistant strains also revealed that resistance is associated with chromosomal genetic elements and extra-chromosomal (plasmid) genetic material which produced genes such as pmrA, pmrB, Alm-EFG, PEA transferase (EPT-ABC), PETN, MSBB, CARR, MCR-1 and mcr-2. Such resistance genotypes are potentially traceable to misuse of antibiotic without prior antibiotic susceptibility testing of isolates. It can also be deduced from the reports that various sources (outbreak, acute diarrhea, environmental, fruit, and vegetables) have been examined which revealed PB sensitive O1 El Tor *V. cholerae* strains. These have further affirmed the prevalence and epidemiological relevance of reports.

In some other related studies, it was reported that a high biotyping and serotyping capability was achieved by phage typing and phage sensitivity which reveals the relevance of phage typing in the characterization scheme [73]. An antibiotic stewardship in the post antibiotic era is suggestive and recommended.

### 4.1 Limitations and strength of the study

The major strength of this study anchors on being the first of its kind in the pool of prevalence and sensitive/resistance status of PB among *V. cholerae* in clinical and environmental nexus. In addition, it also reveal the emerging/dual/atypical and variant nature of the *V. cholerae* strains as it affects the biotyping scheme. The poor determination of PB resistant genes and the non-reporting after a positive *in vitro* susceptibility testing is a notable limiting factor of the study. Another limitation is the exclusion of non-English language full text articles/documents which has to a greater extent affected the exhaustiveness of documents applied for the study. In addition, the exhaustive retrieval of PB sensitive/resistance studies might not be possible as most investigators only report in part some of the resistance observed in their studies while other study focus on non-English language. Other intrinsic indices may also create a bias for the meta-analysis results and interpretation.

Although there are observable potential and strengths of the current study which applied the PRISMA guidelines, there had been some limitations. It also employed the critical appraisal of study quality, analysis of heterogeneity using specified models, inclusion and exclusion criteria etc. In addition, the study also shows the dwindling nature of the biotype characterization scheme as observed from diverse studies in various continents. However, some other limitations include inability to recover documents/articles from such a large continental regions such as Asia, low representative estimates of prevalence and epidemiological relevance on isolates from regions

## 5 Conclusion

This study systematically meta-analyzed and evaluated the status of PB sensitive/resistance among clinical and environmental *V. cholerae* strains using secondary data from global investigators published reports in relevant databases. Such reports suggest that the biotype classification scheme using the resistance and/or susceptibility to PB may not be an appropriate strategy for biotyping the family of Vibrionaceae as there has been in reports the undulating biotype nature of such strains. The use/application of further molecular biological strategies for monitoring the levels of viable PB sensitive and viable PB resistant *V. cholerae* strains in both surface and deep water nexus, environment and public health systems possess a potential in monitoring the emerging tendency of the global menace of cholera. Some of such may include the use of loop-mediated isothermal amplification-polymerase chain reaction (LAMP-PCR), random amplified polymorphic DNA-polymerase chain reaction (RAPD-PCR), etc.

In addition, our results has also revealed evidences on the presence of potential classical and El Tor strains of *V. cholerae* both in the environment and clinical specimens affirming the potential for the spread/distribution of such strains via the aforementioned routes. This suggests the need for adroit surveillance scheme as well as an updated biotyping scheme. The observation of PB -sensitive strains may be spreading in various continents of the world which compromises the several decades-long biotyping scheme and may possibly necessitate the modification and/or reversal of the biotype classification scheme. There is need to carefully monitor and holistically evaluating the clinical and epidemiological relevance of the disseminating new potential variant strains which is endemic in different localities.

## Additional materials

S2 text.

## Appendix Supplementary materials

Image, application 1
