# Supplementary material for: Polymyxin sensitivity/resistance cosmopolitan status, epidemiology and prevalence among O1/O139 and non-O1/non-O139 Vibrio cholerae: A meta-analysis
Source: Infect Med (Beijing). 2023 Nov 21;2(4):283–93. doi: 10.1016/j.imj.2023.11.004 (PMC10774663; doi:10.1016/j.imj.2023.11.004)
Supplement: Supplementary file 1 [file mmc1.docx]

**Supplementary files**


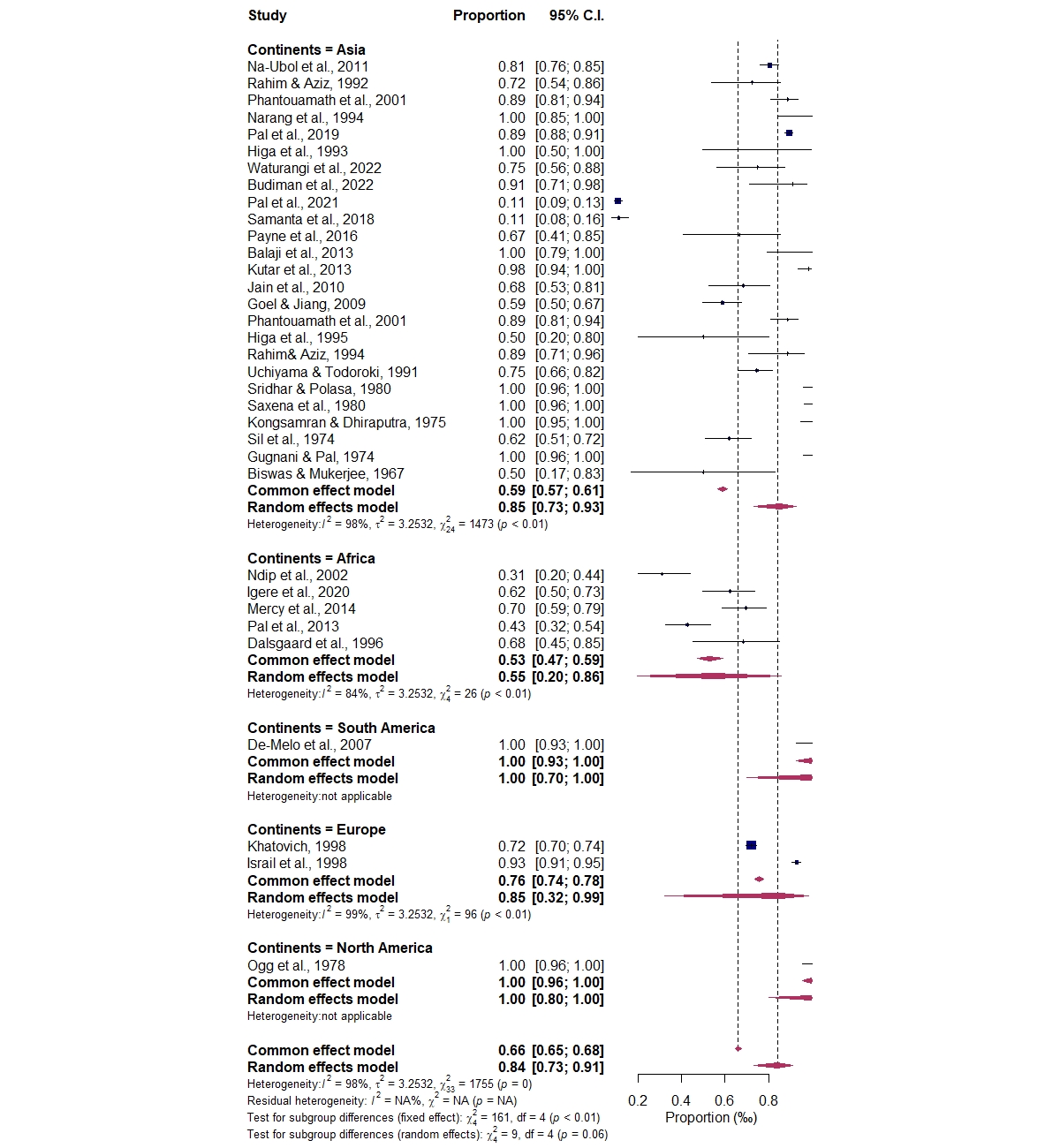


**Figure S1. Continent subgroup analysis on prevalence of polymyxin resistance in *V. cholerae***


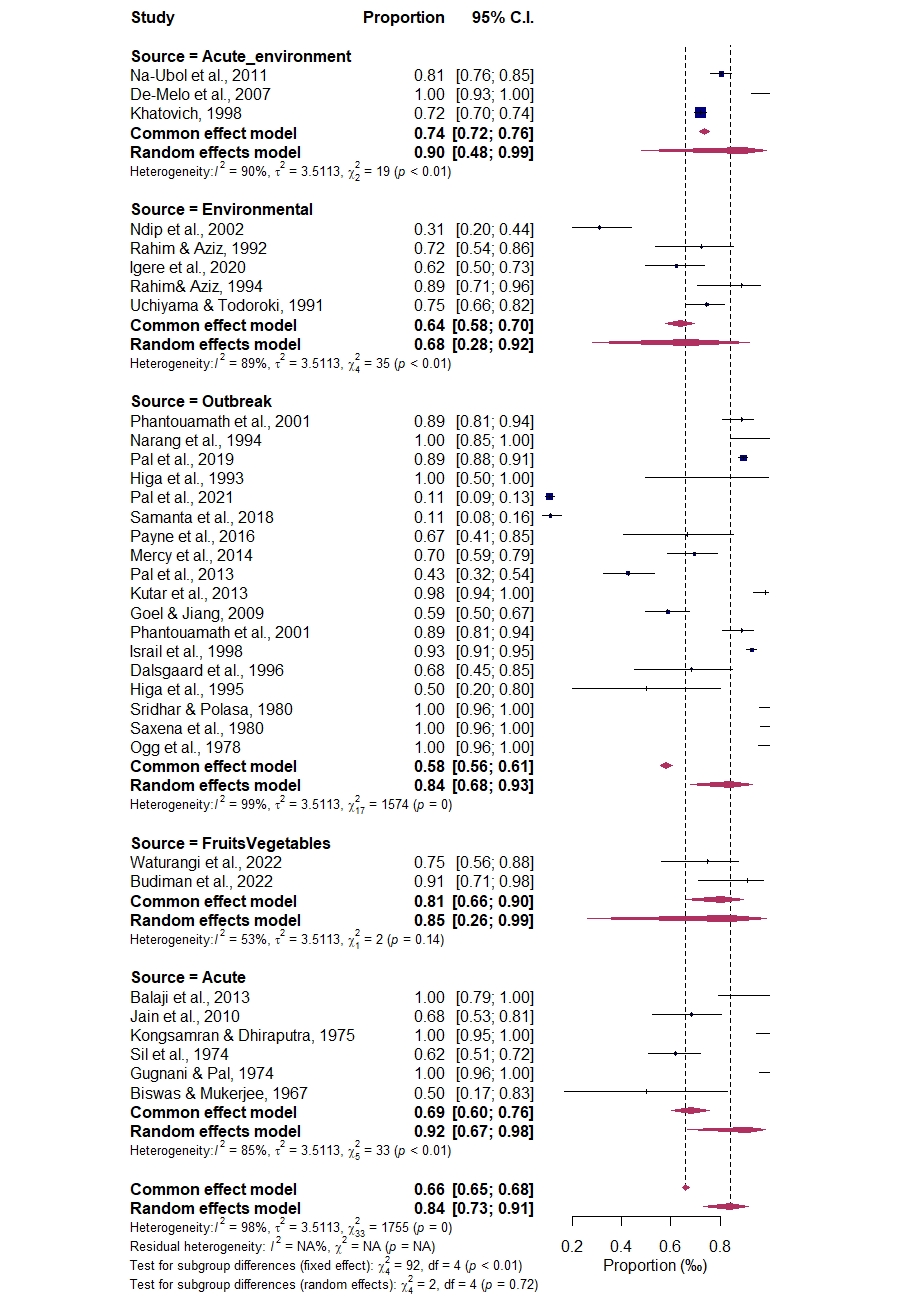


**Figure S2. Sources subgroup analysis on prevalence of polymyxin resistance in *V.* cholerae.**


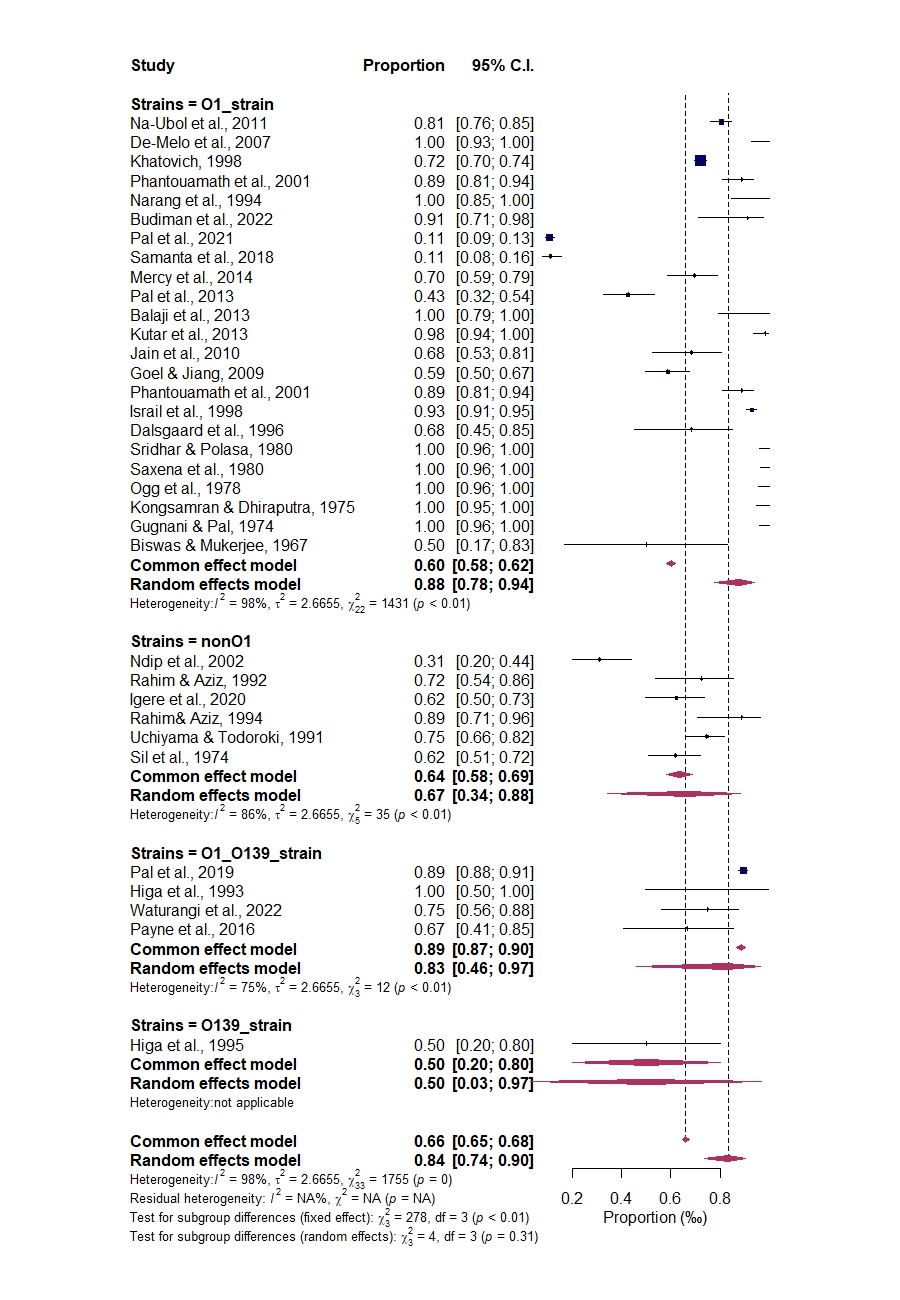


**Figure S3. Strains subgroup analysis on prevalence of polymyxin resistance in *Vibrio* cholerae.**


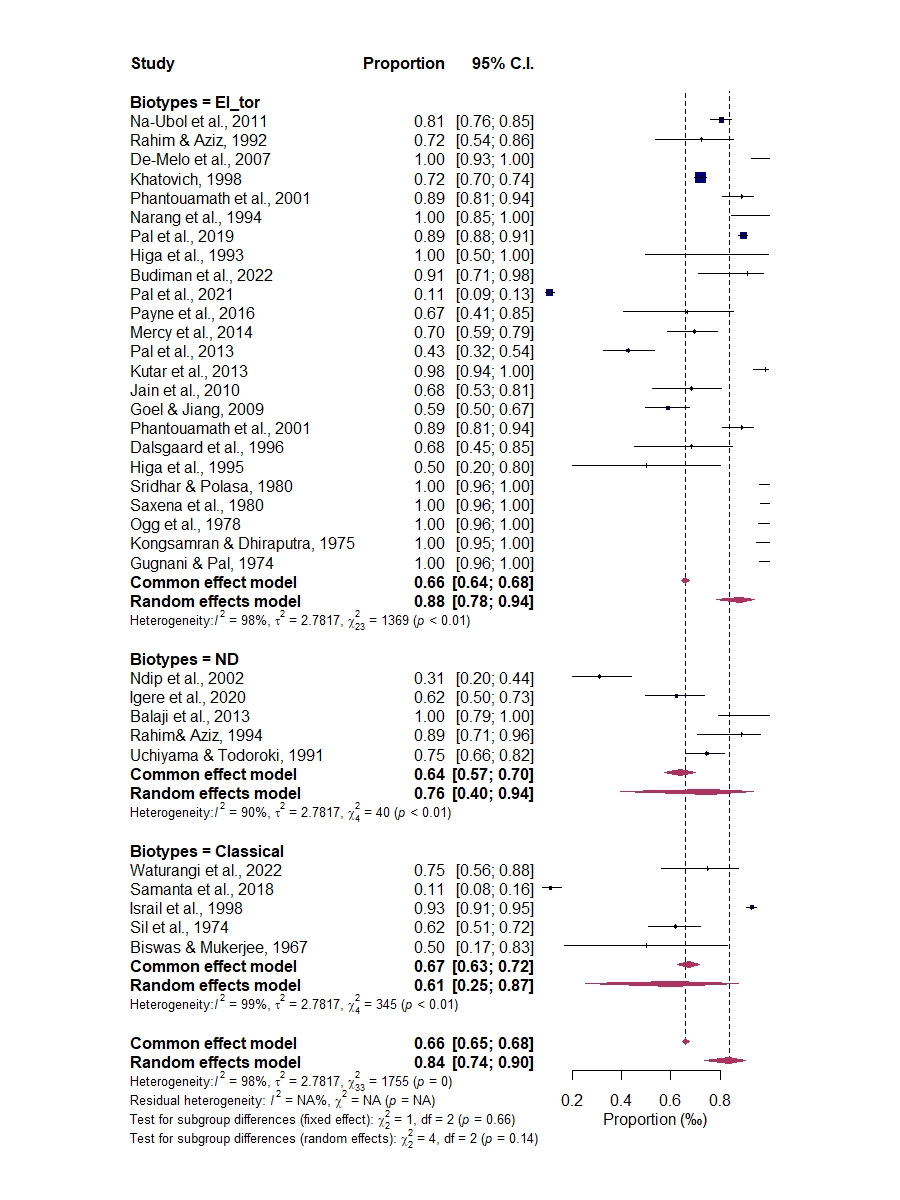


**Figure S4. Biotypes subgroup analysis on prevalence of polymyxin resistance in *Vibriocholerae.***

**Table S1a: Quality assessment of the included studies based on Newcastle-Ottawa Scale (NOS)**

| Authors | Year of Isolation /reporting | Item 1 | Item 2 | Item 3 | Item 4 | Item 5 | Item 6 | Item 7 | Item 8 | Item 9 | Item 10 | Item 11 | Total score | Level of bias |
| --- | --- | --- | --- | --- | --- | --- | --- | --- | --- | --- | --- | --- | --- | --- |
| NA-UBOL et al. | 2011 | 2 | 2 | 2 | 2 | 2 | 2 | 1 | 2 | 2 | 2 | 2 | 21 | low |
| NDIP Rnet al. | 2002 | 2 | 2 | 2 | 2 | 1 | 1 | 2 | 2 | 2 | 2 | 2 | 20 | low |
| RAHIM & AZIZ | 1994 | 2 | 2 | 2 | 1 | 1 | 1 | 2 | 2 | 2 | 2 | 2 | 19 | low |
| DE MELO et al. | 2007 | 1 | 2 | 2 | 2 | 1 | 1 | 1 | 1 | 1 | 1 | 1 | 14 | Moderate |
| KHATOVICH | 1998 | 2 | 2 | 1 | 2 | 1 | 1 | 2 | 2 | 2 | 2 | 2 | 19 | low |
| PHANTOUAMATH et al. | 2001 | 2 | 2 | 1 | 2 | 1 | 1 | 2 | 2 | 2 | 2 | 2 | 19 | low |
| NARANGet al. | 1994 | 2 | 1 | 1 | 2 | 1 | 1 | 2 | 2 | 1 | 2 | 2 | 17 | Moderate |
| PAL et al. | 2019 | 2 | 2 | 2 | 1 | 2 | 1 | 1 | 2 | 1 | 2 | 1 | 17 | Moderate |
| HIGAet al | 1993 | 2 | 2 | 2 | 2 | 2 | 2 | 1 | 2 | 2 | 2 | 2 | 21 | low |
| WATURANGI et al. | 2022 | 2 | 2 | 2 | 2 | 1 | 1 | 2 | 2 | 2 | 2 | 2 | 20 | low |
| IGERE et al. | 2020 | 0 | 1 | 2 | 1 | 2 | 2 | 2 | 2 | 2 | 2 | 2 | 18 | low |
| PAL et al. | 2021 | 2 | 2 | 2 | 2 | 2 | 2 | 2 | 2 | 1 | 2 | 2 | 21 | low |
| BUDIMAN et al. | 2022 | 2 | 2 | 2 | 2 | 2 | 1 | 2 | 2 | 2 | 1 | 2 | 20 | low |
| SAMANTAet al. | 2018 | 2 | 2 | 2 | 2 | 1 | 1 | 2 | 2 | 1 | 2 | 2 | 19 | low |
| PAYNE et al. | 2016 | 2 | 2 | 2 | 2 | 2 | 2 | 2 | 2 | 2 | 2 | 2 | 22 | low |
| MERCY et al. | 2014 | 2 | 2 | 2 | 1 | 2 | 1 | 2 | 2 | 2 | 2 | 2 | 20 | low |
| PAL et al. | 2013 | 2 | 1 | 2 | 2 | 1 | 1 | 2 | 2 | 2 | 1 | 2 | 18 | low |
| BALAJI et al. | 2013 | 2 | 2 | 2 | 1 | 1 | 1 | 2 | 2 | 2 | 1 | 1 | 17 | Moderate |
| JAIN et al. | 2013 | 2 | 2 | 2 | 2 | 1 | 1 | 2 | 2 | 2 | 2 | 1 | 19 | low |
| KUTAR et al. | 2013 | 2 | 2 | 2 | 1 | 2 | 2 | 2 | 2 | 2 | 2 | 2 | 21 | low |
| GOEL and JIANG | 2009 | 2 | 2 | 2 | 1 | 1 | 1 | 2 | 2 | 2 | 2 | 2 | 19 | low |
| PHANTOUAMATHet al. | 2001 | 2 | 2 | 2 | 1 | 1 | 1 | 1 | 2 | 2 | 2 | 1 | 17 | low |
| ISRAIL et al. | 1998 | 2 | 2 | 1 | 2 | 2 | 2 | 1 | 2 | 2 | 2 | 1 | 19 | low |
| DALSGAARD et al. | 1996 | 2 | 2 | 2 | 2 | 2 | 2 | 2 | 2 | 2 | 2 | 2 | 22 | low |
| HIGA et al. | 1995 | 2 | 2 | 2 | 2 | 2 | 2 | 2 | 2 | 2 | 2 | 2 | 22 | low |
| RAHIM and AZIZ | 1994 | 2 | 2 | 2 | 1 | 1 | 1 | 2 | 2 | 2 | 2 | 2 | 19 | low |
| UCHIYAMA and TODOROKI | 1991 | 2 | 2 | 2 | 1 | 1 | 1 | 2 | 2 | 2 | 2 | 2 | 19 | low |
| SRIDHAR and POLASA | 1980 | 2 | 2 | 2 | 1 | 2 | 2 | 2 | 2 | 2 | 1 | 2 | 20 | low |
| SAXENA et al. | 1980 | 2 | 2 | 2 | 1 | 1 | 1 | 2 | 2 | 2 | 2 | 2 | 19 | low |
| OGG et al. | 1978 | 2 | 2 | 2 | 2 | 2 | 2 | 2 | 2 | 2 | 2 | 2 | 22 | low |
| KONGSAMRAN and DHIRAPUTRA | 1975 | 2 | 2 | 2 | 2 | 2 | 2 | 2 | 2 | 2 | 2 | 1 | 21 | low |
| SIL et al. | 1974 | 2 | 2 | 2 | 2 | 2 | 2 | 2 | 1 | 2 | 2 | 1 | 20 | low |
| GUGNANI and PAL | 1974 | 2 | 2 | 2 | 2 | 2 | 2 | 1 | 2 | 2 | 2 | 2 | 21 | low |
| BISWAS and MUKERJEE | 1967 | 2 | 2 | 2 | 2 | 2 | 2 | 2 | 2 | 2 | 2 | 2 | 22 | low |

**Keyword for total score interpretation of bias level: Total score >18 = low; 14 – 18 = moderate ; <13 = high**

**Table S1b: Quality assessement Response Instrument for nonhuman-based prevalence studies**

| **To assess risk of bias related to the study purpose, data collection, method applied and statistical soundness of nonhuman-based prevalence studies** | | | |
| --- | --- | --- | --- |
| Major Components | Response options | | |
| 1. Does the title speaks directly to the present subject being addressed? | No (0 point) | Somehow (1 point) | Yes (2 point) |
| 2. Was the aim of study clearly stated and is it relevant to the present study | Unclear and irrelevant (0 point) | Clearly stated but some how relevant (1 point) | Clearly stated and relevant (2 point) |
| 3. Do results reported in abstract and/or summary similar/matched with those ones presented within the text? | Unmatched (0 point) |  | Matched (2 point) |
| 4. Was the sampling plan description adequate, accompany with date and sample handling information? | No (0 point) | Somehow but inadequate (lack sampling period /sample handling information) (1 point) | Adequately reported with sampling period and sample handling information (2 point) |
| 5. Was the sample size appropriately stated or computable from reported data of the study? | No (0 point) | Somehow but inadequate (1 point) | Yes (2 point) |
| 6. Was the sample size adequate enough (≥50arbitrary selected)? | <50 (0 point) | ≥50or <100 (1 point) | ≥100 (2 point) |
| 7. Was the bacterial density, concentration, recovery or extraction procedure adequately reported and accompanied with a control sample? | Not reported (0 point) | Somehow but with no positive control (1 point) | Reported and adequate with positive control (2 point) |
| 8. Was the bacterial detection assay described in details and reproducible? | No (0 point) | Somehow but with no positive control (1 point) | Yes and with positive control (2 point) |
| 9. Does the endpoints address the study focus and appropriate for the achievement of the study aim and objectives? | No (0 point) | Somehow but inadequate (1 point) | Yes and adequate (2 point) |
| 10. Does the method employed and assessed enhance the study endpoint (outcome) qualitatively or quantitatively in an unbiased manner? | No(0 point) | Somehow but inadequate (1 point) | Yes and adequate (2 point) |
| 11.Was the choice of statistical analysis adequate for the study? | Not reported (0 point) | Somehow but inadequate (1 point) | Yes and adequate (2 point) |

**Keyword for total score interpretation of bias level: Total score >18 = low; 14 – 17 = moderate; <13 = high**
